# Fault Diagnosis Method for Centrifugal Pumps in Nuclear Power Plants Based on a Multi-Scale Convolutional Self-Attention Network

**DOI:** 10.3390/s25051589

**Published:** 2025-03-05

**Authors:** Chen Li, Xinkai Liu, Hang Wang, Minjun Peng

**Affiliations:** 1 College of Nuclear Science and Technology, Harbin Engineering University, Harbin 150001, China; lchen@hrbeu.edu.cn (C.L.); heulxk@hrbeu.edu.cn (X.L.); heupmj@hrbeu.edu.cn (M.P.); 2 Fundamental Science on Nuclear Safety and Simulation Technology Laboratory, Harbin Engineering University, Harbin 150001, China

**Keywords:** centrifugal pump, acoustics and vibration signals, fault diagnosis, deep learning, multi-scale hybrid feature

## Abstract

**Highlights:**

- A multi-scale convolutional self-attention (MS-CSA) method is proposed to improve the accuracy of fault diagnosis for rolling bearings in nuclear power plants.
- This paper designs a multi-scale hybrid feature idea to enrich the fault information present in the features.
- Verification tests were conducted based on sound and vibration experimental data, and the results showed that the fault diagnosis model based on the multi-scale convolutional self-attention (MS-CSA) method significantly improved its diagnostic performance.

**Abstract:**

The health status of rotating machinery equipment in nuclear power plants is of paramount importance for ensuring the overall normal operation of the power plant system. In particular, significant failures in large rotating machinery equipment, such as main pumps, pose critical safety hazards to the system. Therefore, this paper takes pump equipment as a representative of rotating machinery in nuclear power plants and proposes a fault diagnosis method based on a multi-scale convolutional self-attention network for three types of faults: outer ring fracture, inner ring fracture, and rolling element pitting corrosion. Within the multi-scale convolutional self-attention network, a multi-scale hybrid feature complementarity mechanism is introduced. This mechanism leverages an adaptive encoder to capture deep feature information from the acoustic signals of rolling bearings and constructs a hybrid-scale feature set based on deep features and original signal characteristics in the time–frequency domain. This approach enriches the fault information present in the feature set and establishes a nonlinear mapping relationship between fault features and rolling bearing faults. The results demonstrate that, without significantly increasing model complexity or the volume of feature data, this method achieves a substantial increase in fault diagnosis accuracy, exceeding 99.5% under both vibration signal and acoustic signal conditions.

## 1. Introduction

Nuclear power plants (NPPs) are intricate engineering systems characterized by technology intensity, complex structures, high costs, and potential hazards. In the event of an accident, the consequences and impacts would be severe. Rotating machinery is one of the most critical components of mechanical equipment in nuclear power plants, encompassing centrifugal pumps, steam turbines, fans, bearings, and more. The health status of rotating machinery in nuclear power plants is vital for ensuring the overall normal operation of the plant system. As typical active equipment, centrifugal pumps are extensively used in nuclear power plants, including key equipment such as main coolant pumps in the primary circuit and main feedwater pumps, circulating water pumps, and condensate pumps in the secondary circuit. The primary function of the centrifugal pumps used in nuclear power plants is to convert the mechanical energy provided by the prime mover into the energy of the transported fluid, typically kinetic energy and pressure energy, which primarily relies on rotating components and their rotation for realization. Although the typical components and mechanisms of centrifugal pump machinery in nuclear power plants are similar to those of conventional pumps, the requirements for safety and reliability in their complex operating environments are higher. Significant performance degradation or failure can not only pose significant safety risks to the system but also require extensive downtime for repairs or replacements, resulting in substantial economic losses. Due to the unique nature of their operating environment, nearly one-third of failures in rotary pump equipment are caused by bearing failures, which can lead to severe vibration, noise, and even equipment damage, impacting the safe operation of nuclear power plants.

In practical engineering, to ensure the normal operation of centrifugal pump equipment, the maintenance strategy commonly adopted in current nuclear power plants is preventive maintenance. This requires operation and maintenance personnel to monitor and conduct periodic disassembly inspections and repairs, which not only wastes significant human and material resources but also cannot effectively guarantee the operational safety of rolling bearings in centrifugal pump equipment, posing considerable safety risks to the operation of nuclear power plants. Therefore, it is necessary to conduct research on fault diagnosis methods for rolling bearings in centrifugal pump equipment, leveraging fault diagnosis techniques to promptly detect early faults and assist operation and maintenance personnel in decision analysis to ensure the safety of nuclear power plants. This addresses the issues of inadequate timeliness, accuracy, and intelligence levels in traditional fault diagnosis methods for rolling bearings in centrifugal pump equipment.

With the relentless advancement of deep learning techniques, their formidable capabilities in deep mining and adaptive learning have progressively liberated fault diagnosis from the constraints of manual prior knowledge, effectively enhancing the nonlinear mapping ability between feature data and fault types. Notable methods in this domain include stacked autoencoders, recurrent neural networks, and convolutional neural networks (CNNs). Zhang et al. proposed a CNN model augmented with data augmentation and anti-training interference capabilities, capable of achieving high-precision fault diagnosis without requiring noise reduction processing [1]. To enable fault classification of hydraulic axial piston pumps through a deep CNN model, Tang et al. transformed signals into time–frequency images using continuous wavelet transforms and then extracted effective features from these images for fault diagnosis using the CNN model [2]. In an effort to mitigate overfitting issues when using CNNs for fault diagnosis, Kumar et al. proposed an improved CNN for the fault diagnosis of centrifugal pumps. By analyzing acoustic emission signals processed with wavelet transforms and introducing an entropy divergence function, they effectively avoided redundant activations in the hidden layers of the CNN, ensuring feature sparsity to prevent overfitting [3]. Zhong et al. focused on the fault diagnosis of cracks in the rotating machinery of NPPs and found that the integrated learning model exhibits superior anti-noise performance compared to the single model [4]. Liu et al. refined a convolutional neural network (CNN) to effectively extract the spatiotemporal features of input data from NPPs. The refined model demonstrates enhanced transfer capabilities and higher application value [5]. Wang et al. developed a fault diagnosis model for rotating machinery in NPPs based on a Deep Convolutional Neural Network (DCNN), which can effectively extract transferable features under different operating conditions and improve the accuracy of cross-domain fault diagnosis [6]. Dao et al. proposed a CNN-LSTM model based on a Bayesian optimization method, which adaptively selects model hyperparameters. The experimental validation indicated that this method outperforms CNN, LSTM, and CNN-LSTM models in fault diagnosis [7]. Qin et al. introduced a Two-Channel Convolutional Neural Network (TC-CNN) model that extracts deep features through one-dimensional and two-dimensional convolutions, concatenates and fuses them, and further classifies faults using SVM. The experimental results demonstrated good fault diagnosis outcomes and robustness [8]. Shan et al. proposed a bearing fault diagnosis method based on acoustic features and deep learning. This method re-extracts Mel-Frequency Cepstral Coefficients (MFCCs) using a CNN to fully obtain high-dimensional abstract features characterizing bearing faults, thereby achieving rolling bearing fault diagnosis [9]. Based on the above achievements, we can see that many scholars have achieved high-precision fault diagnosis using methods such as stacked autoencoders, recurrent neural networks, and CNNs. This demonstrates the powerful data mining and adaptive learning capabilities of deep learning methods, greatly eliminating dependence on prior knowledge and significantly improving the nonlinear mapping ability between mining feature data and fault types. However, with the deepening development of technology, the emergence of self-attention mechanisms in recent years has brought new directions for fault diagnosis.

In recent years, with the popularization of self-attention mechanisms, many studies have combined them with other deep learning algorithms to selectively focus on fault features containing critical information, overcoming the limitations of single deep learning methods. Cheng et al. proposed an Adaptive Fault Attention Residual Network (AFARN) based on physical information. This model can simultaneously utilize fault features and label information to train the model, aligning diagnostic-relevant feature distributions and providing interpretability [10]. Hu et al. presented a fault diagnosis model combining EfficientNet and a self-attention mechanism, accurately establishing relationships between fault features and fault modes under limited resources and achieving optimal diagnostic performance [11]. Jiang et al. developed an adaptive sparse attention network to analyze fault signal features in different frequency bands of rolling bearings under various fault types, achieving commendable overall performance in cross-condition diagnostic accuracy and model convergence speed [12]. Li et al. introduced an Attention Improved CNN (AT-ICNN) fault diagnosis method that combines CNN and attention mechanisms. By incorporating Improved CNN (IMConv) and an integrated hybrid attention mechanism, it effectively extracts relevant fault information, enhancing the model’s ability to highlight fault features and capture global information [13]. Tang et al. considered bearing vibration signal characteristics and proposed a Trusted Multi-scale Quadratic Attention-Embedded CNN (TMQACNN), which performs well under noise interference or varying loads [14]. Xia et al. proposed a Hierarchical Attention-Based Multi-Source Data Fusion Method (HAMFD) for fault diagnosis, which assesses and identifies faults through multi-layer attention distributions, achieving high accuracy and success rates in fault identification [15]. Xin et al. introduced a Deep Multitask-Based Multi-scale Feature Fusion Network Model (MEAT) to address the limitations and poor adaptability of traditional CNN models for complex tasks [16]. Zhang et al. proposed a Novel Dual Attention Mechanism Network (DAMN), which exhibits favorable diagnostic accuracy and model convergence speed in various fault scenarios [17]. Zhong et al. introduced a Parallel Learning Attention-Guided Convolutional Neural Network (PLA-CNN) that couples a noise reduction model with a fault diagnosis model, effectively enhancing the accuracy of fault identification with the assistance of the noise reduction model [18]. Zhou et al. designed a CNN fault diagnosis method based on a frequency attention mechanism. Compared to the convolutional block attention mechanism, the method combining spatial and channel attention mechanisms improves accuracy by 0.34% and 0.24%, respectively [19]. Wang et al. proposed a fault diagnosis method that combines 1D-CNN with attention mechanisms and hyperparameter optimization. By integrating CNN with attention mechanisms to enhance fault feature information, they also introduced a population optimization algorithm to solve the problem of model hyperparameter selection [20]. Yan et al. proposed a lightweight fault diagnosis framework named LiConvFormer, designed to address the issue of excessive complexity in collaborative models that integrate Transformers and CNNs. The experimental results indicate that this fault diagnosis model framework achieves notable advancements in terms of both lightweight performance and robustness [21]. Dong et al. proposed a one-dimensional improved self-attention-enhanced convolutional neural network (1D-ISACNN) for rolling bearing fault classification. This model demonstrates high classification accuracy across three diverse bearing datasets and exhibits superior recognition precision and stronger generalization capabilities compared to other models [22]. Zhou et al. proposed a deep convolutional generative adversarial network (DCGAN) for semi-supervised fault detection and diagnosis in gear systems. It achieves high diagnosis accuracy with scarce labeled data and extends the capability to diagnose unseen faults beyond the training dataset, validated through systematic case studies [23]. Xiong et al. proposed a new dimensionless indicator, CEMDI, which combines CEEMD with MDI for fault diagnosis in petrochemical units. The proposed method, utilizing CNNs and GAFs, demonstrates superior performance in identifying fault types under various conditions compared to traditional and latest published methods [24].

Based on the above research, we can find that many studies construct fault diagnosis models by integrating self-attention with convolutional networks. Being able to selectively focus on fault features containing key information overcomes the limitations of a single deep learning method and achieves excellent performance in multiple validation sets. These advances indicate that attention mechanisms have great potential to improve the accuracy of fault diagnosis and model performance. However, current diagnostic models typically use single-scale convolution kernels to extract fault features, which makes it difficult to capture complete and detailed information present in the fault features, resulting in the loss of some fault information and affecting the accuracy of fault diagnosis. Yu et al. designed a deep transfer learning model for bearing fault diagnosis, which integrates time–frequency analysis, ResNet, and the self-attention mechanism. The proposed optimization approach fully leverages the advantages of high-dimensional spatial distribution difference evaluation and gradient reversal adversarial strategies. In tasks involving transfer fault diagnosis under varying operating conditions, this model demonstrates superior performance compared to other intelligent fault diagnosis methods, enhancing the cross-domain invariance and fault state discrimination capabilities of deep features [25]. Liu et al. proposed an imbalanced fault diagnosis approach that combines an improved multi-scale residual generative adversarial network (GAN) with a feature enhancement-driven capsule network. The proposed method effectively processes imbalanced fault data, achieving enhanced performance, stability, and diagnostic accuracy compared to state-of-the-art methods [26]. Chen et al. proposed an automatic feature learning neural network that uses raw vibration signals to automatically extract frequency signal characteristics using two convolutional neural networks. The proposed method achieves 98.46% average accuracy, outperforming state-of-the-art intelligent algorithms and demonstrating better performance in noisy environments [27]. Wang et al. proposed a novel deep transfer learning model called the multi-scale deep intra-class adaptation network, which extracts and analyzes low-level features at multiple scales for fault classification. The proposed method achieves high-precision diagnosis results across 24 transfer learning experiments, demonstrating its reliability and generalizability under different working loads [28]. The fault diagnosis approach based on multi-scale feature fusion technology integrates features from diverse scales, utilizing the detailed information contained in shallow features to supplement the deep features. This method effectively mitigates the deficiencies of traditional single-scale analysis methods in comprehensively extracting fault characteristic information of bearings under complex conditions.

Therefore, this paper focuses on the rolling bearings of circulating water pumps in nuclear power plants. We conduct fault simulation experiments on these bearings to acquire acoustic fault signals and extract fault features. Subsequently, we propose an intelligent fault diagnosis model for rolling bearings based on the multi-scale convolutional self-attention (MS-CSA) method. Firstly, time domain signal analysis is performed on vibration signals and acoustic signals based on signal processing methods to extract time domain fault features that characterize the signal fault state. Then, through the deep feature extraction network of the autoencoder, the goal is to enhance the fault representation ability of deep features and combine shallow and deep features to construct a multi-scale mixed feature set, enriching the fault information present in the features. Finally, by combining convolutional neural networks with self-attention mechanisms, a nonlinear mapping relationship between the mixed fault feature set and faults was established. The research outcomes presented in this paper effectively enhance the feasibility of applying intelligent fault diagnosis technology to the fault diagnosis of rolling bearings in circulating water pumps in nuclear power plants, thereby avoiding unnecessary reactor shutdowns and excessive periodic maintenance, thus ensuring the operational safety of nuclear power plants while improving their operational economy.

The structure of this paper is organized as follows: Section 1 introduces the significance of rolling bearings in rotating machinery within nuclear power plants, as well as the importance and challenges associated with fault diagnosis research in this context. Section 2 presents preliminary content, including convolutional neural networks, attention mechanisms, and autoencoders. Following this, it delves into the proposed methodology, encompassing deep and shallow feature extraction techniques, the construction of multi-scale hybrid features, and the fault diagnosis model. In Section 3, the design of the experimental setup and the methodologies for extracting sound and vibration signals are detailed. Subsequently, Section 4 presents the experimental results and discussions under sound and vibration conditions. Finally, Section 5 concludes this paper with a summary of the findings.

## 2. Materials and Methods

### 2.1. Convolutional Neural Network (CNN)

Convolutional neural networks (CNNs) consist of multiple layers, including convolutional layers, pooling layers, and fully connected layers. The input data are sequentially propagated through these layers, with the output of each layer serving as the input to the next. After passing through several convolutional and pooling layers, the data ultimately go through the fully connected layers to produce the final output.

The convolution operation is the fundamental computational procedure within the convolutional layers. The parameters involved in the convolution calculation include the input, weights, and biases, and its complete computational expression is shown in the following equation:

(1) $$y_{ij}=f\left(\sum_{i=1}\sum_{j=1}x_{ij}w_{ij}+b\right)$$

In the equation presented above, $y_{ij}$ represents the output of the convolution operation, $x_{ij}$ denotes the input to the convolution operation, $w_{ij}$ stands for the weight values, *b* signifies the bias term, and *f (*·*)* designates the nonlinear activation function.

The computational process of the convolutional layer is expressed by the formula as follows:

(2) $$Y_{z}^{i}=f\left(\sum_{z=1}^{N}W_{z}^{i}\times Y_{z}^{i-1}+b_{z}^{i}\right)$$

In the equation above, $Y_{z}^{i}$ denotes the *z* feature map of the *i* convolutional layer, where *z* represents the number of convolution kernels in the z convolutional layer. *f (*·*)* is the nonlinear activation function, $W_{z}^{i}$ represents the z convolution kernel in the *i* convolutional layer, and × indicates the convolution operation. $Y_{z}^{i-1}$ represents the *z* feature map from the preceding layer, and *b* denotes the *i* bias in the *z* convolutional layer.

Pooling layers decrease the amount of information that subsequent layers need to process, facilitating the training process and improving the accuracy of the network. Pooling layers are primarily categorized into max pooling and min pooling, with their mathematical models represented by the following equations.

(3) $$\left\{\begin{array}{l} y_{\max}=\max\left(x_{11},\cdots x_{ij}\right)\\ y_{\min}=\min\left(x_{11},\cdots ,x_{ij}\right) \end{array}\right.$$

In the equation above, *y* represents the pooling output, while $x_{ij}$ denotes the input value at position (*i*, *j*), indicating that the maximum or average value within the pooling region is selected for output.

### 2.2. Attention Mechanism

The attention mechanism can be abstractly conceptualized as a feature selection process constituted by query (Q), key (K), and value (V). This mechanism enables a heightened focus on features that exhibit greater relevance to the task among the input information, while mitigating attention towards less relevant features and potentially filtering them out altogether. Consequently, this enhances the efficiency and accuracy of task processing.

Given an index position denoted as z∈[1,N], and under the conditions of specified *q* and *X*, the probability of selecting the input information, denoted as $\alpha_{i}$, is formulated as follows:

(4) $$\alpha_{i}=p\left(\left.z=i\right|X,q\right)=\mathrm{softmax}\left(s\left(x_{i},q\right)\right)=\frac{e^{s\left(x_{i},q\right)}}{\sum_{j=1}^{N}e^{s\left(x_{j},q\right)}}$$

In the equation above, $X=\left[x_{1},x_{2},\ldots ,x_{N}\right]$ represents a set of *N* input information items, $s\left(x_{i},q\right)$ denotes the attention scoring function, and *q* stands for the query vector, with the objective of selecting certain useful information from *X*. An example of such a scoring function is the scaled dot-product function, which is expressed as follows:

(5) $$s\left(x_{i},q\right)=\frac{x_{i}^{T}q}{\sqrt{d}}$$

In the equation above, *d* represents the dimension of the vector *x*. The purpose of multiplying by $\frac{1}{\sqrt{d}}$ is to address the issue where, as the input dimension becomes large, the value of $x_{i}^{T}q$ also becomes significantly large, causing the softmax function to enter a range with very small gradients.

To further improve the training speed, multiple *q_s_*, *k_s_*, and *v_s_* of the same dimension are grouped together to form matrices *Q*, *K*, and *V*, respectively. When the input is *X*, represented by $Q=XW^{Q}$, $K=XW^{K}$, and $V=XW^{V}$, the corresponding weight matrices for *Q*, *K*, and *V* are denoted as $W^{Q}$, $W^{K}$, and $W^{V}$, respectively. The mathematical expression for the attention function is given as:

(6) $$f\left(Q,K,V\right)=\mathrm{softmax}\left(\frac{QK^{T}}{\sqrt{d_{k}}}\right)V$$

In the equation above, the input consists of *Q* and *K* with dimension *d_k_* and *V* with dimension *d_v_*. The scaled factor, denoted as $\sqrt{d_{k}}$, between *Q* and *K* is computed through the softmax function to obtain the weights for *V*.

### 2.3. Auto Encoder (AE)

**Figure 1:**
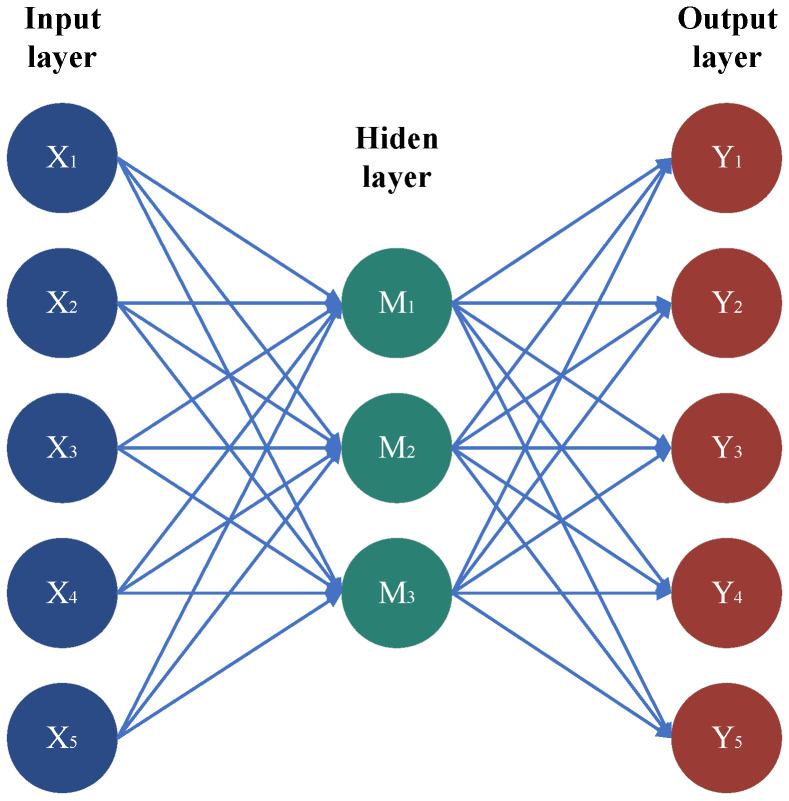
Schematic diagram of the autoencoder structure.

An autoencoder is an un-supervised machine learning algorithm typically composed of an encoder and a decoder. Figure 1 shows a schematic diagram of an AE with a three-layer structure, where the encoder is capable of extracting feature representations from the original signal. The encoder is commonly utilized for dimensionality reduction and feature extraction, with the hidden layer in Figure 1 representing the obtained feature representations. The decoder, on the other hand, is able to transform these feature representations into a reconstructed signal that has the same dimensionality as the original input. 

### 2.4. Multi-Scale Convolutional Self-Attention (MS-CSA)

The design of multi-scale networks has consistently been one of the popular research directions in the field of computer vision. In recent years, numerous researchers have applied multi-scale feature fusion models to recognition, classification, and detection tasks [29,30], achieving promising experimental results. Some traditional network models, which only consider single-scale features, may suffer from inaccurate feature extraction, thereby affecting experimental outcomes. These multi-scale feature fusion methods introduce new ideas to traditional neural network models. By fusing features from different scales, the fused features contain richer information, thereby enhancing model performance.

Convolutional kernels, due to their inherent locality principle, have demonstrated extraordinary ability in capturing low-level features, effectively supporting the initial stage of information processing. The self-attention layer, with its unique global receptive field property, can selectively focus on and emphasize key features even in shallow layers of network architecture. Within the framework of complex network models, the collaborative integration of convolutional kernels and attention mechanisms fully leverages the advantages of these two technologies, which can capture small details while perceiving key features present in global features.

Through the processing of signals collected by sensors, preliminary shallow fault characteristics are initially extracted, which are then subjected to further refinement to obtain deep fault characteristics. These are subsequently integrated to form multi-scale mixed fault features. This approach harnesses the concept of multi-scale networks, effectively capturing the intricate mapping relationships between fault features and modes while simultaneously enriching the feature information through the incorporation of shallow fault characteristics. This methodology not only significantly elevates the diagnostic accuracy of the system by ensuring that the most pertinent features contribute meaningfully to the final outcomes, but it also sustains a rapid training speed, which is crucial for the scalability and practicality of such advanced models.

**Figure 2:**
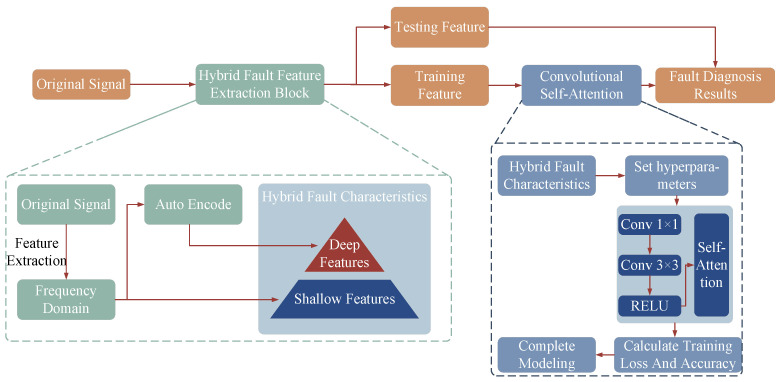
A fault diagnosis model based on the multi-scale convolutional self-attention mechanism Network.

Therefore, this paper proposes a multi-scale hybrid feature complementarity mechanism, which is integrated with a fault diagnosis model constructed based on a CNN and the self-attention mechanism, to establish a fault diagnosis model grounded in a multi-scale convolutional self-attention network. This model amalgamates shallow and deep features to construct a hybrid fault feature dataset, thereby enriching the detailed characteristics of rolling bearing faults. The structure and implementation process of this model are shown in Figure 2. The code library is available at https://github.com/CH9826/M-CSA-Fault-Diagnosis (accessed on 19 December 2024).

The specific implementation process of the fault diagnosis model based on the multi-scale convolutional self-attention mechanism network in this paper is as follows:

1. To fully reveal the inherent patterns and characteristics present in the collected signals from the circulating water pump’s rolling bearings, a shallow feature dataset is constructed using time–frequency domain indicators such as standard deviation, variance, root mean square value, kurtosis, skewness, clearance factor, peak factor, impulse factor, shape factor, information entropy, permutation entropy, and Theil coefficient. This initial step extracts key information that can identify fault characteristics or abnormal states.
2. The shallow features representing the inherent patterns and related characteristics of the rolling bearing signals are further processed through an autoencoder for feature extraction. This generates deep fault features, which are then combined with the aforementioned time–frequency domain features to form a mixed-scale feature set that integrates both deep and shallow features.
3. A multi-scale convolutional self-attention network is constructed by stacking convolutional kernels of different scales with a self-attention mechanism. The local optimality of 1 × 1 and 3 × 3 convolutional kernels is utilized for feature extraction from shallow features. Additionally, the global receptive field of the self-attention mechanism is employed to extract key features from the mixed-scale feature set, aiming to clarify the nonlinear mapping relationship between rolling bearing fault modes and their characteristics.
4. The obtained mixed-scale feature set is used to train the multi-scale convolutional self-attention network. After training, a validation set is utilized to assess the feasibility and effectiveness of the fault diagnosis model. This model can guide the periodic maintenance of nuclear power plants, ensuring operational safety while enhancing economic efficiency.

## 3. Experiment

To simulate typical faults in the rolling bearings of circulating water pumps in nuclear power equipment and analyze their failure mechanisms, this paper establishes a scaled-down experimental test bench for circulating water pumps. Typical fault modes such as inner race fracture, outer race fracture, and pitting corrosion of rolling elements are set up to conduct fault experiments on the rolling bearings of the circulating water pump. The specific process of constructing the experimental test bench and the experimental steps are as follows:

### 3.1. Experimental Test Bench for Rolling Bearing Faults

**Figure 3:**
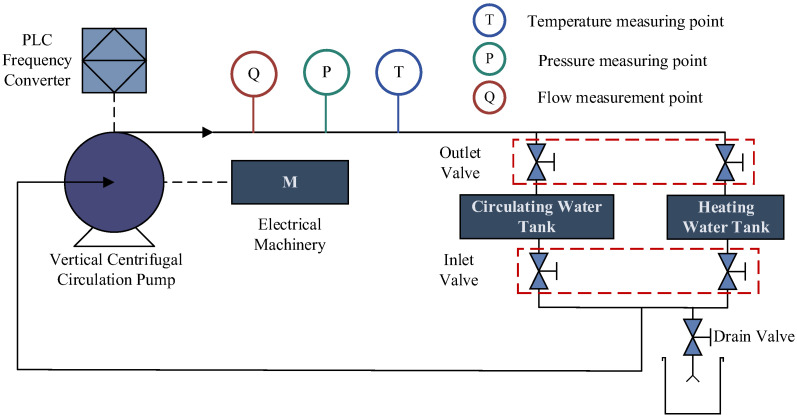
Overall design diagram of the experimental bench for the rolling bearing failure of a circulating water pump.

**Figure 4:**
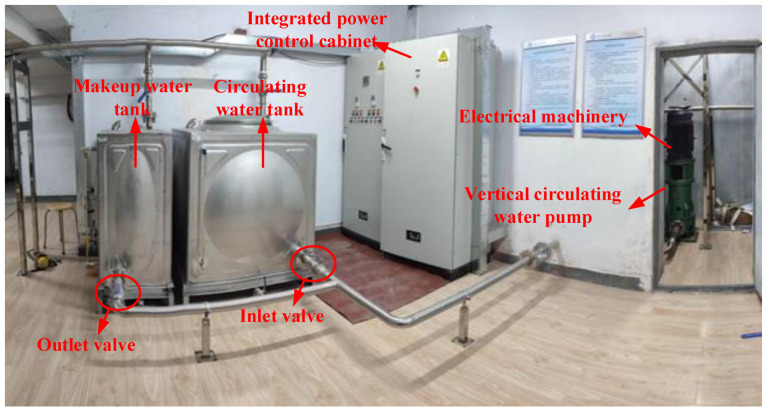
Actual effect diagram of the experimental bench for the rolling bearing failure of a circulating water pump.

The experimental test bench primarily consists of the experimental subject itself and a data acquisition system. The hardware equipment of the test bench mainly includes circulating water pipelines, a motor, a vertical circulating water pump, valves, a water tank, an integrated control platform, and various signal acquisition and measurement devices. The overall scheme design of the experimental test bench is shown in Figure 3, and the actual effect of the test bench is presented in Figure 4. As illustrated in Figure 3, the vertical circulating water pump provides a pressure head for the circulating loop, and the faulty component for the typical fault rolling bearing experiment is located at the lower part of this water pump. To simulate the circulating water system of a nuclear power plant, separate circulating and makeup water tanks are set up to provide the working medium for the loop. The makeup water tank has a heating function, enabling it to simulate temperature variations in the working medium within the loop of a nuclear power plant. Temperature, pressure, and flow sensors are arranged at the inlet and outlet of the vertical circulating water pump to collect parameters such as the temperature, pressure, and flow rate of the working medium in the loop.

### 3.2. Experimental Setup for Rolling Bearing Faults

**Figure 5:**
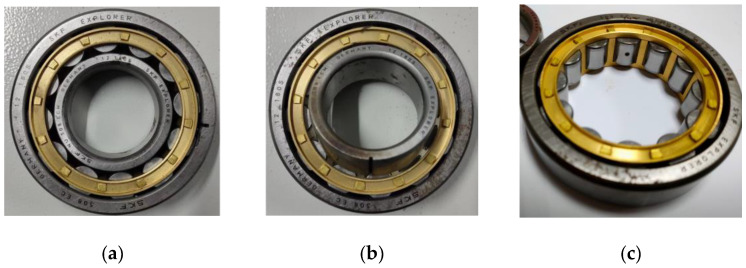
Rolling bearing faulty parts. (**a**) Outer ring fracture fault; (**b**) Inner ring fracture fault; (**c**) Rolling element pitting fault.

The primary faults in the rolling bearings of circulating water pumps are pitting and fracture, which require artificially induced damage on healthy bearings to create faulty components. To simulate early-stage faults with minor crack severity and small pitting diameters, this experiment refers to standard datasets, both domestic and international, taking into account that the fault diameter and depth should not exceed 1% of the outer diameter. Consequently, the pitting-type fault damage diameter and the fracture-type fault damage width are set at 0.9 mm, with a depth of 1.2 mm for both. These damages are induced using wire-cutting techniques to manufacture various faulty components The specific dimensions of the bearings used are presented in Table 1, and an illustration of the faulty rolling bearing components is shown in Figure 5.

#### 3.2.1. Layout of Vibration Signal Measuring Points

**Figure 6:**
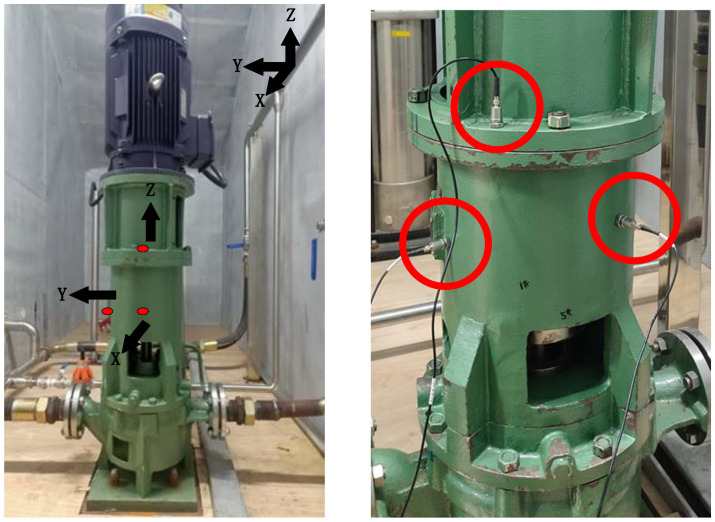
Layout of vibration signal measurement points for the circulating water pump.

In the experiment of a rolling bearing failure in the circulating water pump, acceleration sensors were arranged in two orthogonal directions (+X axis and +Y axis) and in the vertical direction (+Z axis) in the middle of the circulating water pump housing, as shown in Figure 6. The red dots and circles in figures indicate the installation positions of X, Y, and Z-axis sensors.

#### 3.2.2. Layout of Acoustic Signal Measurement Points

**Figure 7:**
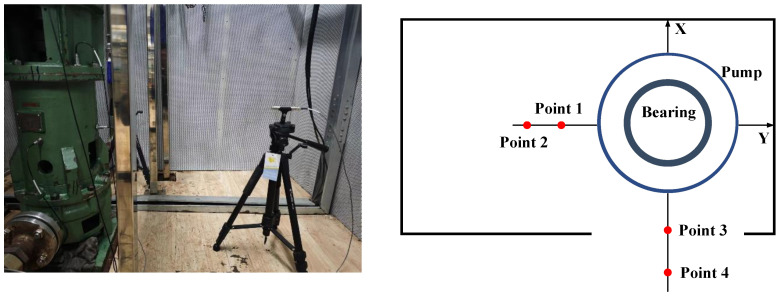
Layout of acoustic signal measurement points for the circulating water pump.

In the experiment of rolling bearing failure in the circulating water pump, two free field microphones were arranged at the X and Y axes orthogonal to the pump body and bearing of the circulating water pump, as shown in Figure 7.

In order to ensure the accuracy of the analysis and minimize the loss of features in the collected signals during the actual experimentation process, it is common practice to set the sampling frequency parameter to be greater than ten times the maximum analysis frequency. Therefore, based on the Nyquist sampling theorem, and drawing from the experimental experience of previous scholars, the sampling frequency of the signals in this experiment was set to *f_s_* = 30,000 Hz, with continuous sampling for 500 s in each set of fault experiments.

According to the above experimental setup, the vibration and acoustic signal data of the rolling bearing under normal operating conditions, outer ring fracture, inner ring fracture, and rolling element pitting conditions are shown in Table 2 below:

## 4. Result Analysis

### 4.1. Experiment Dataset

Based on the above simulation experiments of rolling bearing faults, we obtained vibration and acoustic signals of rolling bearings under normal operating conditions, outer ring fractures, inner ring fractures, and rolling element pitting conditions. However, due to the presence of noise interference in equipment acoustic signals and vibration signals, as well as the large amount of signal data collected, based on simulated experimental data of rolling bearings, this article constructs a shallow fault feature dataset through multi-dimensional feature extraction methods such as time domain, frequency domain, and information entropy. This method can accurately identify and screen indicators that are highly sensitive to fault features and rich in information and ensure comprehensive and accurate capture of fault features. Ultimately, it achieves the effect of improving the accuracy and efficiency of fault feature extraction, providing more reliable input for subsequent fault diagnosis. Therefore, this section adopts the method of dividing windows, setting the window length to 200, to collect the time–frequency domain features contained in different window signals.

The feature data included in the training, testing, and validation sets are presented in Table 3, while the proportions of the training, testing, and validation sets are shown in Table 4.

Among them, the training set and the testing set participate in the model training process, and the training set provides the required parameters for each training step of the model. The test set is used to test the models trained in each step and determine their effectiveness. The validation set does not participate in the training process to ensure the independence of the dataset, which is used to verify the effectiveness of the model obtained through the training process on this dataset.

### 4.2. Vibration Signal Test

**Figure 8:**
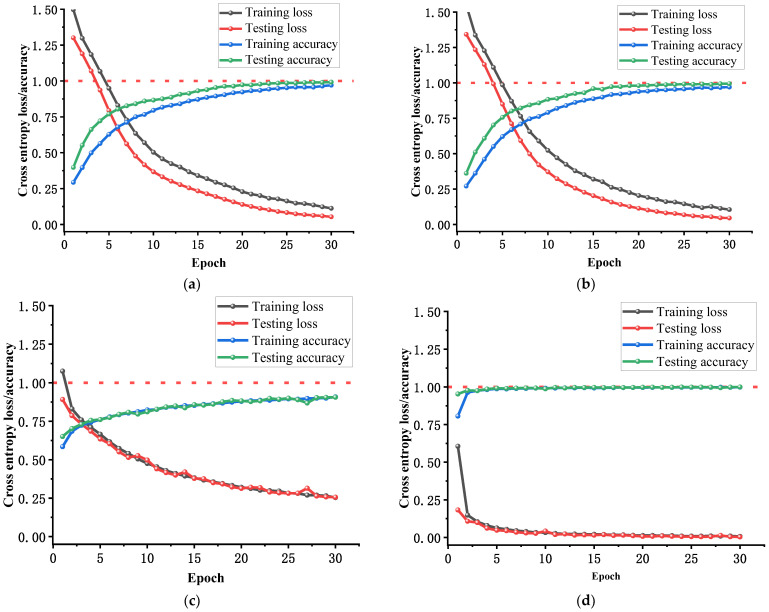
The change curves of training loss, testing loss, training accuracy, and testing accuracy during 30 iterations of four fault diagnosis models by vibration signal: (**a**) CNN, (**b**) TCN, (**c**) CSA, and (**d**) MS-CSA.

**Figure 9:**
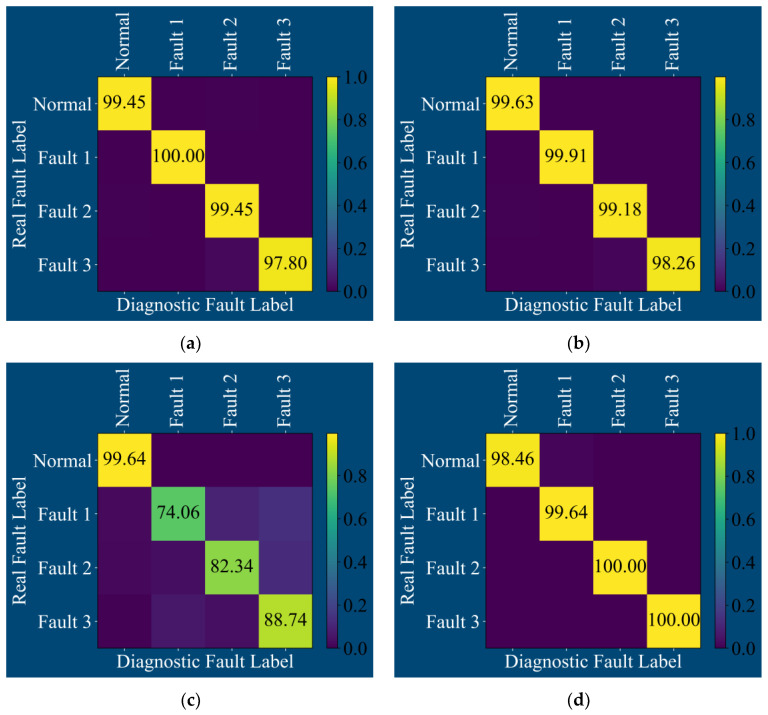
Confusion matrix of four fault diagnosis models by vibration signal: (**a**) CNN, (**b**) TCN, (**c**) CSA, and (**d**) MS-CSA.

After inputting the fault feature sets of vibration signals into the CNN, TCN, the convolutional self-attention network model (CSA), and MS-CSA fault diagnosis models, the training effects are shown in Figure 8 and Figure 9. The structure of the CSA model is the same as that of the MS-CSA model, but CSA only inputs shallow features, while the MS-CSA model inputs mixed fault features.

As can be observed from Figure 8a,b, during the 30 iterations of training, both the CNN and TCN models exhibit a steady increase in training and testing accuracies as the number of iterations increases, gradually approaching 100% accuracy and reaching a state of convergence. The training and testing losses of both models also steadily decrease with the increase in iterations. Figure 8c shows that during the 30 iterations of training, the CSA model’s training and testing accuracies continue to increase with the number of iterations, but there is still some distance from achieving nearly 100% accuracy, indicating that it has not reached complete convergence. Although the training and testing losses of the CSA model also steadily decrease with the increase in iterations, they remain relatively high. As seen in Figure 8d, during the 30 iterations of training, the MS-CSA model achieves a training and testing accuracies of over 75% in the first iteration and immediately reaches an accuracy close to 100% in the second iteration, demonstrating its exceptional convergence ability on the same dataset. Additionally, the training and testing losses of the MS-CSA model reach an excellent performance close to 0 within five iterations. Figure 8 reveals that compared to the CNN, TCN, and CSA models, the MS-CSA model exhibits superior convergence ability and speed.

The confusion matrix output after training the above four models is shown in Figure 9:

From the confusion matrices in Figure 9a,c, it can be observed that the CNN exhibits misdiagnosis for rolling element faults, where some rolling element faults are incorrectly classified as inner race faults. The CSA model also shows misdiagnosis for outer race faults, inner race faults, and rolling element faults. In comparison, as seen from the confusion matrices of the TCN and MS-CSA models in Figure 9b,d, both models demonstrate a relatively clear classification of the four states of the rolling bearings.

**Figure 10:**
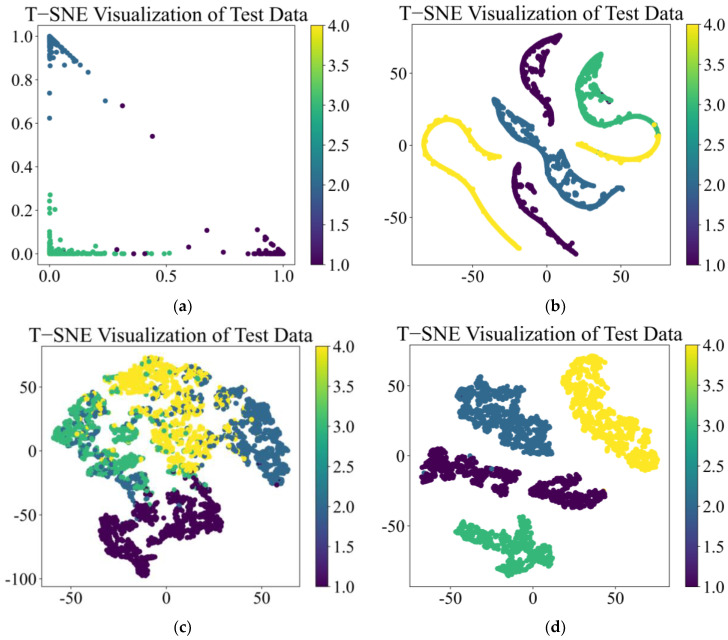
t-SNE of four fault diagnosis models by vibration signal: (**a**) CNN, (**b**) TCN, (**c**) CSA, and (**d**) MS-CSA.

Among them, the training time required for the CSA and MS-CSA models is shown in Table 5:

From Table 5, we can see that the training time of the MS-CSA model improved based on the multi-scale mixed feature mechanism does not significantly increase compared to the training time of the CSA model.

t-Distributed Stochastic Neighbor Embedding (t-SNE) is an algorithm that reduces the dimensionality of complex high-dimensional data. T-SNE creates a reduced feature space, where similar samples are modeled by nearby points and dissimilar samples are modeled by high probability distant points. By using the t-SNE method to reduce high-dimensional complex features to a low-dimensional space and visualize them, data with similar structures will form a cluster of feature points to represent the performance of deep learning models in classification tasks. The t-SNE output after training the above four models is shown in Figure 10:

From Figure 10a,b, it can be seen that the CNN and TCN have excellent feature classification performance under vibration signal conditions and can clearly classify four types of features with almost no overlapping relationship between features. In Figure 10c, CSA performs poorly in feature classification, with large overlapping relationships between features that are difficult to distinguish. In Figure 10d, the MS-CSA model, improved based on the CSA model, has excellent performance in feature classification, with four types of features clearly divided into four clusters.

Table 6 presents the fault diagnosis performance of each model on the validation set after training. The validation set is independent of the training and testing processes of the four models, providing a high level of reliability for assessing the models’ fault diagnosis capabilities. As can be seen from Table 6, both the CNN and TCN models achieved fault diagnosis performance with an accuracy rate above 90% on the validation set. The performance of the CSA model on the validation set was relatively poor, with an accuracy rate above 90% only for the normal and inner race fracture states of the rolling bearings. For the outer race fracture and ball pitting states, the fault diagnosis performance of the CSA model was quite poor, failing to reach an accuracy rate above 90%, with the diagnostic accuracy for outer race fracture being only 82.28%. The MS-CSA model exhibited exceptional performance on the validation set, achieving a diagnostic accuracy rate of 99.5% for all four states of the rolling bearings.

Through the comparative analysis of the training, testing, and validation stages of the four models under vibration signal conditions, it can be observed that the proposed MS-CSA model in this paper demonstrates exceptional model convergence ability, convergence rate, and diagnostic accuracy. Compared to the CSA model, the proposed MS-CSA model, due to its unique multi-scale network model mechanism, combines shallow features from the input with deep features extracted through the autoencoder for training. Without altering the original model structure or increasing the feature magnitude, it significantly enhances the training effectiveness and validation accuracy of the original model.

### 4.3. Acoustic Signal Test

**Figure 11:**
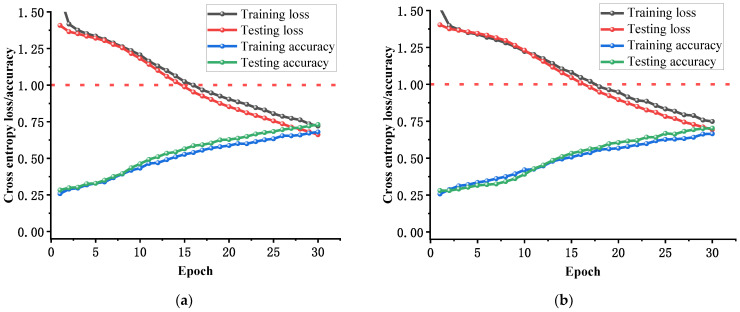

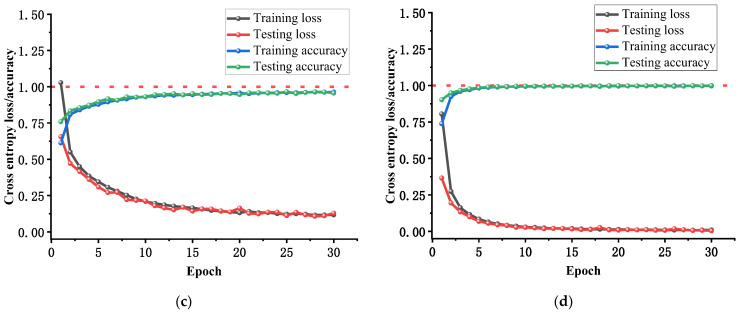
The change curves of training loss, testing loss, training accuracy, and testing accuracy during 30 iterations of four fault diagnosis models by acoustic signal: (**a**) CNN, (**b**) TCN, (**c**) CSA, and (**d**) MS-CSA.

**Figure 12:**
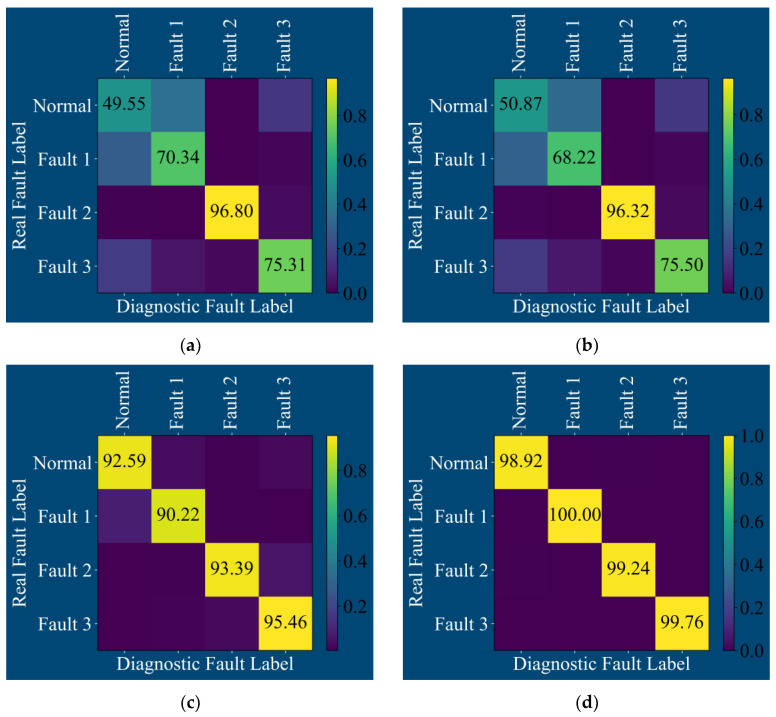
Confusion matrix of four fault diagnosis models by acoustic signal: (**a**) CNN, (**b**) TCN, (**c**) CSA, and (**d**) MS-CSA.

After inputting the acoustic signal fault feature set into the CNN, TCN, CSA, and MS-CSA fault diagnosis models, the training results are shown in Figure 11 and Figure 12. The structure of the CSA model is the same as that of the MS-CSA model, but CSA only inputs shallow features, while the MS-CSA model inputs mixed fault features.

As depicted in Figure 11a,b, during the 30 iterations of training, both the CNN and TCN models exhibit an increasing trend in training and testing accuracies as the number of iterations increases. However, there is still a significant gap before their accuracy approaches 100%, with neither model achieving even 80% accuracy, indicating a lack of model convergence in this dataset. Although the training and testing losses of the CNN and TCN models also steadily decrease as the number of iterations increases, they remain relatively high. Figure 11c illustrates that during the 30 iterations of training, the CSA model demonstrates a steady increase in training and testing accuracies as the number of iterations increases, gradually approaching 100% accuracy and achieving a convergent state. The training and testing losses of the CSA model also steadily decrease as the number of iterations increases. As shown in Figure 11d, during the 30 iterations of training, the MS-CSA model achieves a training and testing accuracies of around 75% in the first iteration and immediately reaches an accuracy close to 100% in the second iteration. This indicates the model’s exceptional convergence ability on the same dataset. Simultaneously, the training and testing losses of the MS-CSA model also exhibit an outstanding performance, approaching zero within five iterations. As evident from Figure 8, the MS-CSA model demonstrates significantly superior convergence ability and speed compared to the CNN, TCN, and CSA models.

The confusion matrix output after training the above four models is shown in Figure 12:

As evident from the confusion matrices in Figure 12a,b, the CNN and TCN models exhibit relatively superior diagnostic performance only for inner race faults. For the three states of normal, outer race fracture, and inner race fracture of rolling bearings, the diagnostic accuracy does not exceed 80%. Even for the normal and outer race fault states, the diagnostic accuracy fails to reach 60%. In comparison, as observed from the confusion matrix of the CSA model in Figure 12c, this model demonstrates fairly good diagnostic performance for the three states of normal, outer race fracture, and ball pitting. However, there is a tendency to misdiagnose inner race fractures of rolling bearings as ball pitting. Looking at the confusion matrix of the MS-CSA model in Figure 12d, this model can provide a clearer classification for all four states of rolling bearings.

Among them, the training time required for the CSA and MS-CSA models is shown in Table 7:

From Table 7, we can see that the training time of the MS-CSA model improved based on the multi-scale mixed feature mechanism does not significantly increase compared to the training time of the CSA model.

**Figure 13:**
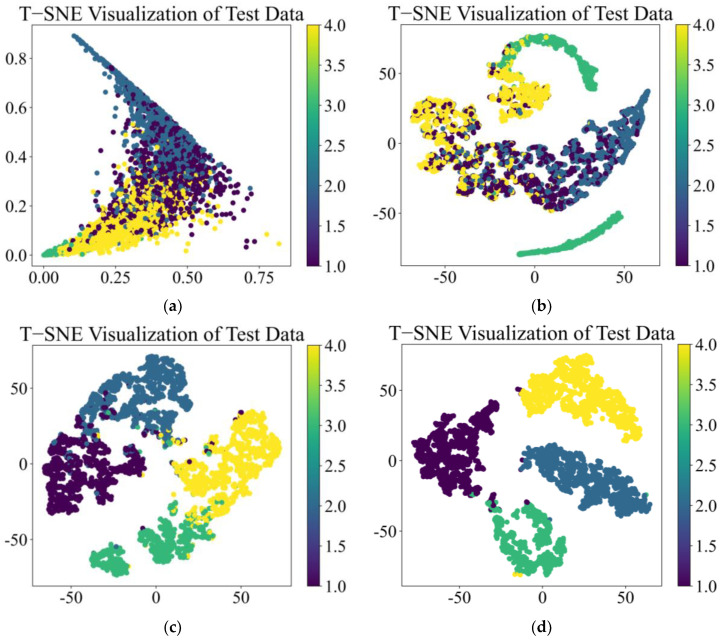
t-SNE of four fault diagnosis models by acoustic signal: (**a**) CNN, (**b**) TCN, (**c**) CSA, and (**d**) MS-CSA.

The t-SNE output after training the above four models is shown in Figure 13:

From Figure 13a,b, it can be seen that CNN and TCN have poor feature classification performance under acoustic signal conditions, with overlapping relationships between the four types of features, making it difficult to perform effective classification. In Figure 13c, CSA performs relatively well under acoustic signal conditions, with only a small portion of features overlapping, making it difficult to distinguish effectively. In Figure 13d, the MS-CSA model, improved based on the CSA model, still has excellent feature classification performance under acoustic signal conditions. The four features are clearly divided into four clusters, with almost no feature overlap.

Table 8 presents the fault diagnosis performance of various models on the validation set after training. Notably, the validation set is independent of the training and testing processes of the four models, offering high credibility for evaluating the models’ fault diagnosis capabilities. As seen from Table 8, the CNN and TCN models exhibit poor performance on the validation set, achieving an accuracy of over 90% only for the inner race fracture state of rolling bearings. The diagnostic accuracy for the remaining three states of rolling bearings does not exceed 75%. The CSA model achieves a diagnostic accuracy of over 90% for the normal, outer race fracture, and ball pitting states of rolling bearings on the validation set. However, its diagnostic accuracy for the inner race fracture fault of rolling bearings reaches 88.75%, which is below 90%. The MS-CSA model demonstrates exceptional performance on the validation set, achieving a diagnostic accuracy of over 99.5% for all four states of rolling bearings.

Through the comparative analysis of the training, testing, and validation stages of the four models under acoustic signal conditions, it is evident that the CSA model exhibits decent performance on this dataset but still encounters misdiagnosis in some states. The MS-CSA model proposed in this paper demonstrates outstanding performance across all four states of rolling bearings and possesses exceptional model convergence capabilities.

### 4.4. Case Western Reserve University (CWRU) Bearing Data Test

**Figure 14:**
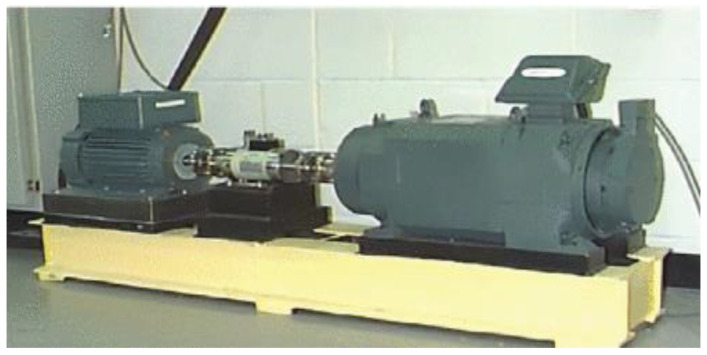
The fault diagnosis test platform of Case Western Reserve University.

To verify the effectiveness of the proposed model, an open-source dataset of rolling bearings was obtained by the fault diagnosis test platform of the petrochemical large-scale rotating machinery at Case Western Reserve University (CWRU), as shown in Figure 14. In the experiment, acceleration data of the driving end at a sampling frequency of 48 kHz were acquired, and the vibration data were obtained by an acceleration sensor mounted on the housing. Each vibration dataset contained 1024 sampling points.

**Figure 15:**
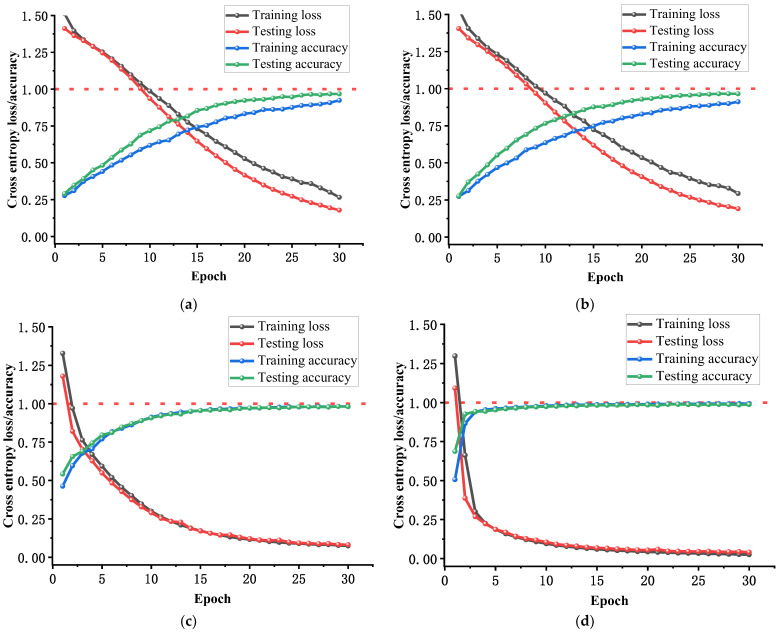
The change curves of training loss, testing loss, training accuracy, and testing accuracy during 30 iterations of four fault diagnosis models by general data: (**a**) CNN, (**b**) TCN, (**c**) CSA, and (**d**) MS-CSA.

**Figure 16:**
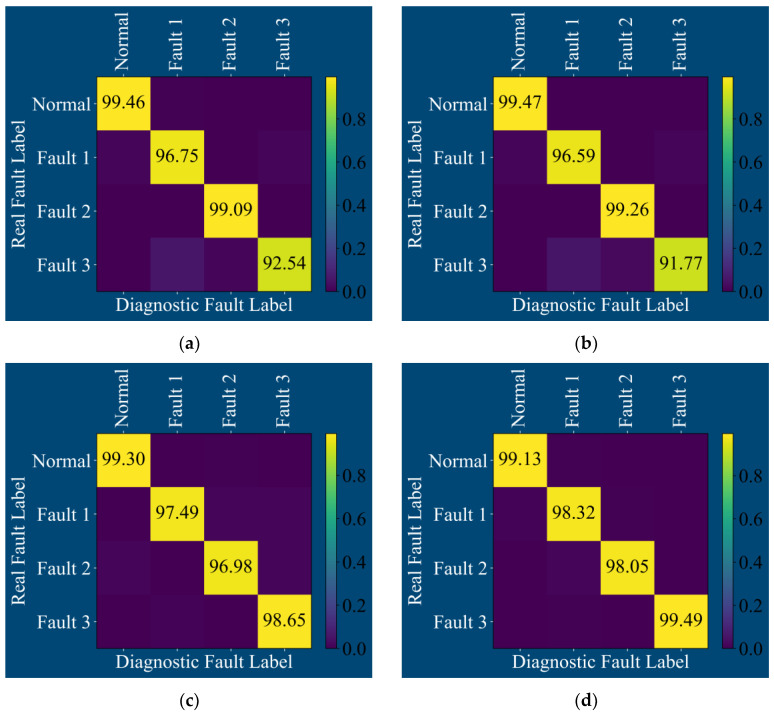
Confusion matrix of four fault diagnosis models by general data: (**a**) CNN, (**b**) TCN, (**c**) CSA, and (**d**) MS-CSA.

After inputting the fault feature set into the CNN, TCN, CSA, and MS-CSA fault diagnosis models, the training results are shown in Figure 15 and Figure 16. The structure of the CSA model is the same as that of the MS-CSA model, but CSA only inputs shallow features, while the MS-CSA model inputs mixed fault features.

As shown in Figure 15a,b, in 30 iterations of training, the training and testing accuracies of both the CNN and TCN models showed an upward trend with the increase in iteration times. However, their accuracy rate increases slowly, and their accuracy only approaches 100% after nearly 30 iterations. Although the training and testing losses of the CNN and TCN models steadily decrease with increasing iteration times, they are still relatively high. Figure 15c shows that during the 30 iterations of training, the CSA model steadily improved in training and testing accuracies as the number of iterations increased, gradually approaching 100% accuracy and reaching a convergence state. As the number of iterations increases, the training and testing losses of the CSA model steadily decrease. As shown in Figure 15d, in 30 training sessions, the MS-CSA model quickly achieved near 100% accuracy in the first few iterations. This indicates that the model has excellent convergence ability on the same dataset. Meanwhile, the training and testing losses of the MS-CSA model also demonstrated excellent performance, approaching zero within five iterations. As shown in Figure 15, compared with the CNN, TCN, and CSA models, the MS-CSA model exhibits significantly superior convergence ability and speed.

The confusion matrix output after training the above four models is shown in Figure 16:

It can be clearly seen from the confusion matrices in Figure 16a,b that the CNN and TCN models can achieve good resolution for four states: normal, outer ring fracture, inner ring fracture, and rolling element pitting. Only the accuracy of diagnosing rolling element pitting is low, but its rotation rate exceeds 90%. As shown in the confusion matrix between the CSA model and the MS-CSA model in Figure 16c,d, both models exhibit excellent diagnostic performance for four states: normal, outer ring fracture, inner ring fracture, and rolling element pitting.

Among them, the training time required for the CSA and MS-CSA models is shown in Table 9:

From Table 9, we can see that the training time of the MS-CSA model improved based on the multi-scale mixed feature mechanism does not significantly increase compared to the training time of the CSA model.

**Figure 17:**
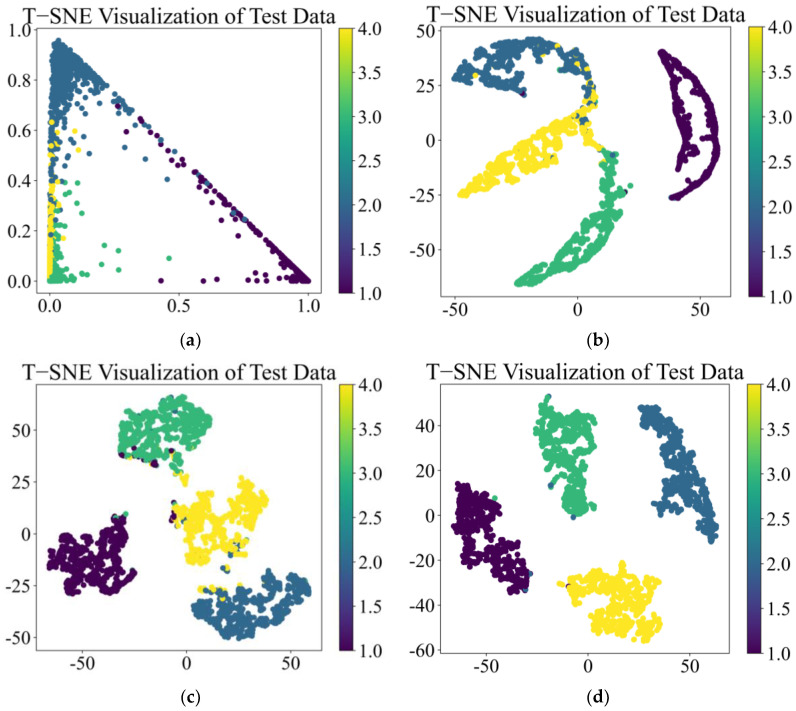
t-SNE of four fault diagnosis models by general data: (**a**) CNN, (**b**) TCN, (**c**) CSA, and (**d**) MS-CSA.

The t-SNE output after training the above four models is shown in Figure 17:

From Figure 17a,b, it can be seen that CNN and TCN have poor feature classification performance under these feature data, with overlapping relationships between features, which may affect the classification performance. From Figure 17c,d, it can be seen that both the CSA and MS-CSA models perform well on these feature data, and these four features are clearly divided into four clusters with almost no feature overlap.

Table 10 shows the fault diagnosis performance of various models on the validation set after training. It is worth noting that the validation set is independent of the training and testing processes of the four models, providing high credibility for evaluating the fault diagnosis ability of the models. From Table 10, it can be seen that the CNN and TCN models perform well on the validation set, with only some scenarios having accuracy rates below 90%. The CSA model and MS-CSA model exhibit excellent performance on the validation set, with diagnostic accuracy exceeding 95% for all four states of rolling bearings. In some scenarios, the accuracy of the MS-CSA model has been improved compared to the CSA model.

Through comparative analysis of the training, testing, and validation stages of four models under acoustic signal conditions, it is evident that the CSA model has shown good performance on this dataset, but misdiagnosis still exists in certain scenarios. The MS-CSA model proposed in this article exhibits excellent performance in all four states of rolling bearings and has outstanding model convergence ability.

## 5. Conclusions

To explore the nonlinear mapping relationship between fault features and fault modes in rotary equipment with pumps, this paper proposes a fault diagnosis method based on a multi-scale convolutional attention mechanism network. The method is validated on datasets of vibration signals and acoustic signal features. The main conclusions are as follows:

1. Traditional convolutional network models such as CNN and TCN perform well in fault diagnosis under vibration signal conditions, but their fault diagnosis performance is not outstanding in acoustic signals with higher noise levels. The CSA constructed by adding an attention module to the traditional convolutional model performs poorly in fault diagnosis under vibration signal conditions but performs better in acoustic signals with higher noise levels.
2. Compared with the three fault diagnosis models, i.e., CNN, TCN, and CSA, the MS-CSA model exhibits better performance in terms of model convergence speed, model convergence capability, and validation set accuracy. This model achieves an accuracy rate of 99.5% for both vibration signals and acoustic signals.
3. Comparing the CSA model with the MS-CSA model, it can be found that combining shallow data-driven models with multi-scale network ideas does not significantly increase model complexity and feature data levels, nor does it significantly increase the training time required. But by combining shallow data-driven models with multi-scale network ideas, the convergence and fault diagnosis capabilities of the original model can be significantly improved, and excellent fault diagnosis performance can be achieved.

In summary, this method enhances the accuracy, reliability, and real-time performance of fault diagnosis for rotating machinery in nuclear power plants. On this basis, the multi-scale network paradigm fully excavates detailed features from the training data, further enriching the nonlinear mapping relationship between fault features and fault modes, and subsequently enhancing the performance of the diagnostic model. This ensures that the method meets the diverse requirements for accuracy and real-time performance in different application scenarios involving vibration signals and acoustic signals of rotating machinery in nuclear power plants.

In our following research, we can further explore the potential of diagnostic methodologies based on multi-scale hybrid features. This approach involves the integration and analysis of features across different scales to comprehensively capture the dynamic behavioral characteristics of rotating machinery within complex operational environments. Furthermore, the concept of multi-scale hybrid features holds considerable application potential in cross-domain diagnostics. By combining common and distinct features among domains through the application of the multi-scale hybrid feature concept, we can enable models to more effectively adapt to fault diagnosis requirements across various domains and operating conditions, thereby enhancing the accuracy and generalization capabilities of cross-domain fault diagnosis. This research direction holds significant theoretical and practical importance and merits in-depth exploration.

## Figures and Tables

**Table 1:** Structural parameters of rolling bearings.

Model	Number of Rolling Elements	Aperture	Outside Diameter	Inner Raceway Diameter
NU 308 ECM	12	40 mm	90 mm	52 mm

**Table 2:** Signal data of the rolling bearing fault simulation experiment.

Signal Type	Rolling Bearing Status	Data Size	Label
Vibration Signal	Normal Bearing Operation	974,848	Normal
	Outer Race Fracture	907,264	Fault 1
	Inner Race Fracture	630,784	Fault 2
	Rolling Element Pitting	1,030,144	Fault 3
Acoustic Signal	Normal Bearing Operation	974,848	Normal
	Outer Race Fracture	907,264	Fault 1
	Inner Race Fracture	630,784	Fault 2
	Rolling Element Pitting	1,030,144	Fault 3

**Table 3:** Fault feature dataset.

Number	Fault Feature
1	Standard Deviation
2	Variance
3	Root Mean Square Value
4	Kurtosis
5	Margin
6	Skewness
7	Peak Factor
8	Pulse Factor
9	Waveform Factor
10	Information Entropy
11	Permutation Entropy
12	Theil Index

**Table 4:** Proportion of training, testing, and validation sets.

	Training Set	Testing Set	Validation Set
Proportion	56%	24%	20%

**Table 5:** Training time required for the CSA and MS-CSA models by vibration signal.

	Training Time (t/s)
CSA	59.21
MS-CSA	62.74

**Table 6:** Accuracy of fault diagnosis for four models by vibration signal.

	Normal	Outer Ring Fracture	Inner Ring Fracture	Rolling Element Pitting Corrosion
CNN	96.57%	91.43%	95.98%	96.25%
TCN	98.65%	93.88%	97.63%	96.25%
CSA	99.18%	82.28%	91.56%	89.21%
MC-CSA	99.90%	99.78%	100%	100%

**Table 7:** Training time required for the CSA and MS-CSA models by acoustic signal.

	Training Time (t/s)
CSA	62.61
MS-CSA	68.68

**Table 8:** Accuracy of fault diagnosis for four models by acoustic signal.

	Normal	Outer Ring Fracture	Inner Ring Fracture	Rolling Element Pitting Corrosion
CNN	55.45%	57.03%	92.41%	72.91%
TCN	55.24%	49.44%	91.11%	74.48%
CSA	98.57%	94.30%	88.75%	97.21%
MC-CSA	99.39%	100%	99.53%	100%

**Table 9:** Training time required for the CSA and MS-CSA models by general data.

	Training Time (t/s)
CSA	61.79
MS-CSA	65.51

**Table 10:** Accuracy of fault diagnosis for four models by general data.

	Normal	Outer Ring Fracture	Inner Ring Fracture	Rolling Element Pitting Corrosion
CNN	89.24%	93.45%	99.36%	99.57%
TCN	88.60%	91.98%	98.95%	94.09%
CSA	99.80%	98.39%	96.57%	97.98%
MC-CSA	99.19%	98.98%	98.25%	100%

## Data Availability

The data presented in this study are available on request from the author.
